# Preservation of human vascular tissue and the relevance of temperature: a narrative review

**DOI:** 10.3389/fbioe.2025.1631214

**Published:** 2026-01-13

**Authors:** Emil-Marian Arbănaşi, Traian V. Chirilă

**Affiliations:** 1 Department of Vascular Surgery, George E. Palade University of Medicine, Pharmacy, Sciences and Technology, Târgu Mureş, Romania; 2 Centre for Advanced Medical and Pharmaceutical Research (CCAMF), George E. Palade University of Medicine, Pharmacy, Sciences and Technology, Târgu Mureş, Romania; 3 Doctoral School of Medicine and Pharmacy, George E. Palade University of Medicine, Pharmacy, Sciences and Technology, Târgu Mureş, Romania; 4 Vascular Surgery Clinic, Mureş County Emergency Hospital, Târgu Mureş, Romania; 5 Faculty of Medicine, George E. Palade University of Medicine, Pharmacy, Sciences and Technology, Târgu Mureş, Romania; 6 Queensland Eye Institute, Woolloongabba, QLD, Australia; 7 Australian Institute of Bioengineering & Nanotechnology (AIBN), University of Queensland, St Lucia, QLD, Australia

**Keywords:** cryopreservation, cryoprotective agents, cryptobiosis, deep-subzero preservation, lyophilization, vascular tissue, vitrification

## Abstract

This review was intended as a conceptual paper exploring the historical background, general principles, and experimental exploits that have steered meaningful developments in the field of temperature-dependent storage procedures and their impact on the attributes and patency of human vascular tissues assigned for use as grafts in cardiovascular surgery or for research purposes. Attention was focused on advances in the field following a descriptive history of humankind’s progress in developing low-temperature methods to conserve and store perishable goods, in understanding cryptobiotic processes and adopting a scientific approach to preservation of biological matter, and in summarizing the pioneering work of Alexis Carrel and others related specifically to the conservation of blood vessels. Further discussed were the principles of low-temperature preservation methods for cells, tissues, and organs, as well as the range of current techniques. The use of particular techniques for the preservation of human vascular tissues, mainly grafts for surgery, was reviewed, emphasizing the extent of their applications, the range of operating conditions (temperature, cryoprotective agents), and the perceived limitations of diverse procedures. It was concluded that many preservation techniques can be employed successfully for storing human blood vessels, however the deep-subzero temperature methods seem to have been the preferred alternative.

## Historical background of conservation at low temperatures

A perennial activity of humankind since the oldest recorded times was to prevent the spoilage of food, especially meat. To that end, methods have been continuously developed, such as cooking, sun drying, smoking, fermenting, or salting. In regions with a warm climate, cooling and freezing also emerged as methods, albeit they implied costly efforts with harvesting the ice and storing it in built pits, cellars, or icehouses, therefore cryopreservation of food and drinks remaining accessible mainly to the privileged classes. Ice also helped people to cool down in the scorching heat, as our species always strived for pleasant living conditions. There is recorded evidence that, 2,000–4,000 years ago, processing and using ice was an organized human activity in the ancient Mesopotamia (Ellison, 1978; Sasson, 1984; Biot, 1851; Li, 2022). Similar pursuits were recorded in Egypt, India, Greece and Roman Empire (Love, 2009). Interestingly, artificial cooling was proposed in Egypt for medical applications, as described in the Edwin Smith Surgical Papyrus. Written in the period 3000 to 2500 BCE, and re-copied around 1700 BCE, this manuscript is considered one of the most valuable ancient medical texts to ever be brought to light and translated into a modern language (Atta, 1999; Feldman and Goodrich, 1999). In the original text (Breasted, 1930), the process of cooling was mentioned as a stage in certain surgical protocols, most likely as a remedy against inflammation, while the cooling technique was not described.

Coming much closer to our times, it is known that Francis Bacon (1561–1626), a philosopher, jurist and scientist considered one of the creators of the modern world, has shown interest in the application of low temperatures for preservation of meat (Jardine and Stewart, 1998). He was also seeking an explanation of the effects of cooling in general, rather than aiming only at a simple application for food conservation. Freezing temperatures in his experiments were achieved by using snow, ice or ice-salt slurries. Bacon’s interest in low temperatures was part of his hypothesis regarding the prolongation of life by the conservation and induration of tissues caused by exposure to low temperatures. At that time, the term ‘induration’ referred to hardening or congealing, somewhat different from today’s meaning (i.e., thickening and hardening of soft tissues due to inflammatory processes; e.g., sclerosis). One of Bacon’s experiments carried out with an eviscerated chicken carcass, stuffed and packed with snow, might have led eventually to his death as he caught pneumonia during the outdoor experiment (Clark, 1898; Francis Bacon, 1993). The episode was first described (Clark, 1898), based on the information provided by Bacon’s friend, the great philosopher Thomas Hobbes. As the historian Thomas Macaulay later stated, “The great apostle of experimental philosophy was destined to be its martyr” (Macaulay, 1882). Although Bacon himself wrote before his death a letter mentioning the chicken experiment, which “succeeded excellently,” there is still skepticism among historians regarding the veracity of this particular episode. There is no doubt, however, that Bacon was a true pioneer of the conservation of tissues at low temperatures (Jardine and Stewart, 1998).

Robert Boyle (1627–1691), a chemist, physicist and inventor, regarded as the founder of modern chemistry, studied the direct action of low temperatures on vegetal and animal tissues. The results were published in a book (Boyle, 1665), mainly dedicated to his “doctrine” of cold, including aspects like freezing techniques and applications to various liquid substances, expansive force of freezing water, relation water-air during freezing, and thawing processes. He also investigated the freezing of plants, animal flesh and excised animal organs (eyes, livers, brains and tongues), using snow-salt mixtures. Employing occasionally a microscope, Boyle discovered that upon freezing the biological fluids (“alimental juices”) transformed into ice crystals (“icy corpuscles”) and separated from the remaining solid matrix. Showing a remarkable prescient insight, he hypothesized that the ice crystals could cause damage to the tissue, and thus accelerating the decomposition of biological matter during and after thawing. To avoid premature decay, Boyle recommended that the thawing should be done slowly (“by degrees”) in an aqueous medium, rather than direct exposure to external heating.

Notable work on the effect of low temperatures was carried out on living matter by Lazzaro Spallanzani (1729–1799) (Spallanzani, 1776; Sandler, 1973; Bwanga, 1991; Sztein et al., 2018), a biologist and a polymath who contributed to the development of many fields of science including reproductive medicine, cryobiology, paleontology, physiology, and echolocation. He demonstrated experimentally that when exposed to temperatures at which water freezes, the animal and human spermatozoa became motionless, only to become active again when the temperature was raised. It took a century for this remarkable finding to be further investigated by Paolo Mantegazza (1831–1910), who documented that human spermatozoa could be frozen at subzero temperatures (−17 °C was the lowest temperature achieved by him), and still be revived upon warming (Sandler, 1973; Bwanga, 1991; Mantegazza, 1866). A true visionary, Mantegazza proposed and predicted the existence of sperm banking for artificial fertilization (Bwanga, 1991; Sztein et al., 2018); it took almost another century for this proposition to become reality (Watson, 1979).

Paul Bert (1833–1886), a French surgeon and physiologist carried out extensive work on the grafting of tissues, limbs and organs in animals (Seghers and Longacre, 1964). The experiments were published as two successive doctoral theses, where he also included his study of the effect of exposing preoperatively the grafts to low temperatures (Bert and De la Greffe, 1863; Bert and De la Vitalité Propre des Tissus, 1866). In general, the lower was the storage temperature, the more successful was the outcome of grafting. At the lowest temperature tested by Bert (−18 °C) (Bert and De la Vitalité Propre des Tissus, 1866), no damage to the tissue was seen. He described hundreds of animal experiments, also attempting to make sense of the knowledge accumulated, and argued for applying grafting to human patients. Unfortunately, the account of his work was buried in two doctoral theses with poor dissemination among peers, while Bert himself abandoned soon afterwards the scientific career to become a politician.

At the beginning of the 20th century, considerable activity was reported in Europe and USA regarding the implantation of blood vessels, which triggered an interest in preservation of arterial or venous *ex-vivo* segments needed for grafting. The foremost representative of this era was Alexis Carrel (1873–1944), one of the few genuine accomplished surgeon-scientists of all times, who modernized vascular surgery and set the groundwork for future human organ transplantations. In 1912, he was the first surgeon to be awarded a Nobel Prize in medicine. His experiments with animal vascular tissues (Carrel, 1907; Carrel, 1908) showed that grafts stored in a refrigerating unit, at temperatures between 0 °C and 4 °C for up to 35 days, led to optimal postoperative outcomes. Rather surprisingly, Carrel also reported (Carrel, 1908) that the grafts frozen for a few days prior to surgery did not perform well in the long-term, probably because of the damage caused by the growing ice crystals. Balanced salt solutions, isotonic to blood plasma, were employed preferentially as preservation media. In a noteworthy experiment (Carrel, 1912a), he removed a segment of popliteal artery from the leg of a human patient and preserved it for 24 days in an isotonic solution at ∼0 °C before transplanting it into the abdominal aorta of a dog. The animal lived in good health for over 4 years when it died from an unrelated cause. He evaluated a large number of freezing media, concluding that isotonic solutions and petrolatum (Vaseline) were the most successful regarding the surgical outcome, within storage periods as long as 1 year at temperatures between −3 °C and 15 °C, usually employing the range 0 °C–1 °C.

Carrel developed a hypothesis regarding the preservation of tissues between collection and implantation stages (Carrel, 1910; Carrel, 1912b). He postulated the existence of two forms of life, *latent* and *active*. In turn, latent life involved two different conditions. The “unmanifested actual life” is the condition where metabolism is reduced almost to cessation, but not completely, a stage called by Carrel “general” death; although it can occur suddenly, the general death is a temporary condition that slowly advances to total breakdown of cellular protoplasm and “elemental” death. He defined the other condition, the “potential” life as the suspension of all vital processes, e.g., metabolism, growth, motility etc. This condition could prevent general death and allow preservation of explanted tissues “outside of the body for an indefinite period of time” (Carrel, 1912b). Going further, Carrel emphasized that the other form of life, the active life, can be maintained for a tissue isolated from a living organism, as demonstrated in 1910 by KeshishianHarrison’s (2004), *if* normal nutrition was provided through artificial means. Carrel’s experiment (Carrel, 1912b) with an isolated chicken’s heart confirmed that crucial finding. His message will remain forever with his successors: the vascular tissue for transplantation must be alive if we aim at a satisfactory outcome.

In current terms, latent life takes effect when an organism enters a reversible ametabolic state called cryptobiosis (Keilin, 1959; Wright, 2001). It is a state of suspended animation that can be caused by dessication (anhydrobiosis), low temperatures (cryobiosis), lack of oxygen (anoxybiosis), high salt concentration (osmobiosis), or high levels of metabolic toxins (chemobiosis). Research on cryptobiosis began centuries ago with the experimental observations on cryobiosis (Power, 1664) and anhydrobiosis (Needham, 1743; Baker, 1764; Mercier Dupaty, 1788) of nematodes. Employing microscopic techniques, van Leewenhoeck (Wright, 2001; Van Leewenhoeck, 1678; Hoole, 1807) carried out investigations on the anhydrobiosis of protists, bacteria, and rotifers. It appears that when formulating his ideas, Carrel was aware of much of that previous work.

The subsequent impressive expanse of research on cryobiology and cryopreservation, and on ensuing applications, were summarized in some excellent reviews (Keilin, 1959; Wright, 2001; Luyet and Gehenio, 1940; Smith, 1958; Bojic et al., 2021; Freitas-Ribeiro et al., 2022; Chen et al., 2023; Arav and Natan, 2024; Khaydukova et al., 2024). It was estimated (Coriell et al., 1964) that by 1940 over 4,000 publications had been already dedicated to the biological effects of subzero temperatures. While significant success was achieved through the development of improved methods based on controlling the freezing/cooling and thawing/warming processes, and by finding better cryoprotective agents, the progress was slower in the field of transplantation of large organs (Bennett, 2025).

## Cryodamage to cells and tissues

Cryopreservation, known also as cryogenic preservation, is an effective method for the long-term storage of cell suspensions, excised tissues, and small or large whole organs. However, its success can be substantially affected by injury to cells and tissues caused by the freezing/cooling and thawing/warming processes. Indeed, as water is omnipresent within biological systems, its solidification into ice crystals within intracellular or extracellular space is fatal to the cell. Therefore, knowledge of water-to-ice phase transition at subzero temperatures and its effect on cytoplasm, cell membrane, and cellular interactions is crucial for finding means to reduce the lethality associated with cryopreservation techniques. Our understanding of the effects of ice’s presence in biological systems, the mechanism of cryodamage, and also the role of cryoprotectants has been summarized in some earlier landmark reviews and in more recent publications as well (Wood et al., 1956; Trump et al., 1965; Mazur et al., 1972; Mazur, 1984; Toner et al., 1990; Karlsson and Toner, 1996; Asghar et al., 2014; Fu et al., 2022; Pegg et al., 2015; Best, 2015; Elliott et al., 2017; Eskandari et al., 2020; Chang and Zhao, 2021; Murray and Gibson, 2022; Landecker, 2024).

A brief description of cryogenic processes based on such publications may be useful in this context. While refrigeration of biologic matter can be employed successfully in certain situations, its major drawback is the limited shelf life of the stored specimens. Cryopreservation at subzero temperatures, e.g., from −130 °C to −196 °C (the boiling point of liquid nitrogen) extends the shelf life to hundreds of years; for instance, a shelf life of a thousand years was estimated theoretically for cells stored in liquid nitrogen (Mazur, 1984). However, the interval from −15 °C to −60 °C during the cooling stage, and passed again during thawing, induces lethal damage to cells due to ice formation. In principle, the damage due to freezing (cryodamage) can be caused by (a) the ice crystals destroying mechanically the cells by perforating and shredding their structure, or (b) secondary effects due to changes in the concentration of solutes in the liquid phase. Both mechanisms are relevant, and the distribution of their inputs is controlled by factors such as cell type, and cooling rate. For instance, at fast cooling, formation of damaging intracellular ice crystals is favored, while large osmotic forces are generated contributing to the rupture of cell membrane. At slow cooling, extracellular ice formation is favored, and the resulting ice crystals cause physical damage to cells, while cells are also subject to prolonged exposure to high concentrations of harmful solutes, both intracellularly and extracellularly. An ideal cooling rate has to be low enough to avoid intracellular ice but high enough to reduce solute effects. The fact that each cell type has a different cooling rate versus survival profile complicates the process if tissues and full organs are involved. Regarding the thawing/warming rate, although the rapid procedures appear to be preferred, it may lead sometimes to cell survival lower than provided by a slower rate. Ice crystal can increase in size (recrystallization) and induce cellular damage and lysis.

All mentioned events could be partially alleviated by the addition of cryoprotective agents (CPAs), also known as cryoprotectants. They have been introduced in methodology in order to reduce or prevent cryoinjury, mainly through diminishing ice formation by lowering the concentration of solutes. The classic cryoprotectants such as glycerol and glycols (Polge et al., 1949) and dimethyl sulfoxide (DMSO) (Lovelock and Bishop, 1959) have limitations, yet DMSO is still routinely employed. As they become common components in most cryopreservation protocols, the CPAs lost the initial novelty, and it was suggested that their actual involvement might have been overlooked (Landecker, 2024). In fact, all elements in the trinity *freezer/target/cryoprotectant* are equally essential. Extensive research is currently carried out to develop more effective CPAs including ice binders, nucleation inhibitors, bio-inspired agents, and substances that may display protective effects even if based on mechanisms not fully elucidated (Murray and Gibson, 2022). CPAs can be either permeating (e.g., DMSO), or non-permeating (e.g., sucrose, trehalose). They are commonly mixed with a vehicle solution, such as RPMI 1640 culture medium, Dulbecco’s modified Eagle’s medium (DMEM), fetal bovine serum, human albumin, or Krebs-Henseleit buffer (KHB) solution. The CPA-vehicle solution mixtures are known as *cryomedia*.

Vitrification is an alternative method for preservation that allows cooling the cells to cryogenic temperatures without the formation of ice crystals (Pegg et al., 2015; Rall and Fahy, 1985; Fahy et al., 2015). The non-freezing aqueous solutions hosting the biological targets solidifies by fast cooling, leading to enormous viscosities (∼10^15^ cP), when the medium turns into a glassy or vitreous material devoid of ice. In this context, the terms ‘freezing’ and ‘thawing’ should be replaced by, respectively, ‘cooling’ and ‘warming’ (or ‘rewarming’). While involving the use of high concentrations of CPAs, vitrification is generally regarded as a successful technique that may display some advantages when compared to other cryopreservation procedures. However, the trade-off between the toxicity and concentration of CPAs remains a challenge. An accurate account of vitrification’s fundamentals and methodology is available (Fahy et al., 2015).

Tissues and organs are systems comprising densely packed cells and extracellular matrix (ECM) components, all assembled with a specific architectonics in order to generate integrated functional entities. To maintain the ECM organization and intercellular relationships following a preservation procedure is crucial. The application to multicellular systems of cryopreservation techniques specific to single-cell systems provides in general disappointing results, especially for organs. Upon freezing, extracellular ice crystals are produced that can damage indirectly the cells and directly the host as such, in which case they may also lead to functional damage. Multicellular systems contain a collection of cell types with variable survival responses to freezing, yet all these types are subjected to the same cooling and thawing processes. Therefore, larger concentrations of CPAs are needed to broaden the survival range. In such systems, the mechanisms of cryodamage become more complex at molecular and ultrastructural levels. Other factors that contribute to cryodamage in organs include non-uniform freezing or thawing rates, anomalous heat and mass transport processes, uneven expansion and contraction leading to fracture, and uneven distribution of CPAs.

We should mention that in organs that requires their own intact vasculature for securing a successful transplantation, the cryodamage ensues through the rupture of capillaries regardless of how many cells survived. The ice forms intraluminally, and rupture occurs when its volume overcomes the limits maintained through the original strength and elasticity of the wall. That may be relevant for vessels larger than capillaries.

## Brief outline of preservation techniques

Normothermic and subnormothermic preservation techniques involve normal temperatures, more precisely near the physiologic temperature (36 °C–38 °C), or in the range 20 °C–33 °C, respectively. The process of cryobiosis is not involved in these procedures, as they do not aim at suspending the metabolism in order to avoid degradation of the preserved biological material, and therefore are practicable only for very short periods of storage. For an improved viability of stored cells, methods based on encapsulation within biocompatible gels have been developed, however the storage time remains limited. For the normothermic and subnormothermic preservation of organs, machine perfusion techniques, pioneered by Carrel and Lindbergh (Carrel and Lindbergh, 1935), have been further significantly refined and are used occasionally in the transplantation of kidney, liver or heart. The principle of perfusion-assisted preservation is to duplicate the original physiological milieu of the organ by combining oxygen-carrier substances, nutrients, antithrombotic drugs, and other agents.

A significant number of cryopreservation techniques are now available, and developmental work is in continual progress aimed at improvements. It is important to mention that the implementation of these methods is associated with unavoidable technical and economical drawbacks, such as dependence on continuous electricity supply, and requirement for large working spaces to accommodate refrigerators, freezers, back-up freezers, and auxiliary equipment. The procedures employing liquid nitrogen need regular refreshments, such generating significant additional costs.

Based on the available literature (Freitas-Ribeiro et al., 2022; Chen et al., 2023; Arav and Natan, 2024; Khaydukova et al., 2024; Taylor et al., 2019; Criswell et al., 2023; Ozgur et al., 2023), we have summarized below the current storage techniques involving low temperatures.

*Hypothermic method* does not involve subzero temperatures, and is commonly known as ‘cold storage.’ It is entirely based on refrigeration (1 °C–8 °C) and is used for short-term storage, i.e., for a few days, but preferably no longer than a few hours. It is generally applicable when other techniques are unsuccessful for particular biological targets. Hypothermia is based on the slowing effect that a low temperature has on the rate of chemical and biochemical processes, a fundamental natural law. It is routinely used in laboratories for *in vitro* experiments involving cells or tissues that are freshly available for proximal use. It cannot provide a basis for long-term storage and banking of biological material for transplantation, which requires much lower temperatures and longer durations. However, when combined with perfusion, it can be used to preserve organs such as kidneys or heart.

*Lyophilization and preservation in dry state*. Known also as *freeze-drying*, this method is based on a process of dehydration at low temperature, where both anhydrobiosis and cryobiosis may be involved. The technique consists of three stages, including (a) freezing (between −50 °C and −80 °C), (b) primary drying achieved by the sublimation of ice under controlled vacuum, and (c) secondary drying achieved by removing the residual (non-freezable) water at raised temperatures. The resulting desiccated porous products can be stored for years at room temperature, and their original properties can be reinstated on demand by rehydration with an appropriate amount of water while controlling the osmotic stress.

*High-subzero preservation* comprises strategies requiring intermediate subzero temperatures, developed as alternatives both to hypothermic and cryogenic methods. Inspired from the stress-tolerance strategies adopted by some species, e.g., frog, crocodile, squirrel, or dolphin (Taylor et al., 2019), this category includes the techniques listed below, using the current terminology.

- *Supercooling* (−4 °C to −6 °C)
- *Partial freezing* (−5 °C to −20 °C)
- *Equilibrium nonfrozen subzero preservation* (−5 °C to −20 °C); known also as the liquidus tracking method
- *Isochoric subcooling* (−5 °C to −30 °C), known also as the high-subzero isochoric preservation, involves pressurizing while maintaining a constant volume

A range of chemical compounds has been developed to serve as cryostasis agents (‘revival cocktails’) for enhancing the outcomes of the high-subzero methods.

*Deep-subzero preservation* employs temperatures in the range −80 °C to −196 °C, and includes both *traditional* cryopreservation and vitrification techniques, well known and used extensively.

Furthermore, innovative vitrification techniques have been developed lately, including:

- *Isochoric vitrification* (−80 °C to −196 °C), maintaining a constant volume that prevents ice formation
- *Nanowarming–assisted vitrification* (−120 to −196 °C), involving rapid warming with heat-generating nanoparticles
- *Viscosity–controlled vitrification* (−120 °C to −196 °C), using non-Newtonian and rheomagnetic fluids that can prevent ice formation

**FIGURE 1:**
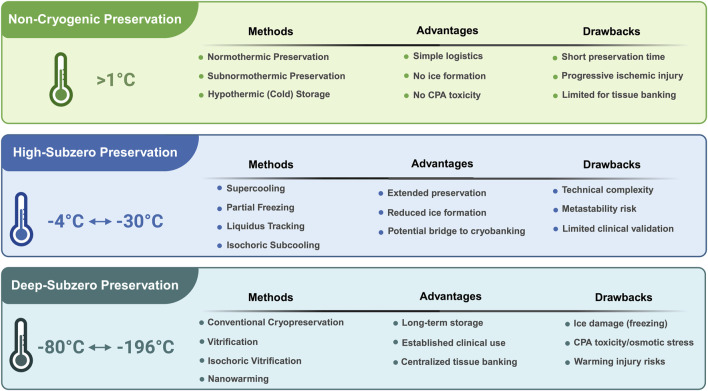
Schematic overview of biological preservation approaches organized by temperature regime. Non-cryogenic preservation (>1 °C) includes normothermic, subnormothermic, and hypothermic storage, offering simple logistics and avoiding ice formation and CPA toxicity, but with limited preservation duration and progressive ischemic injury. High-subzero preservation (−4 °C to −30 °C) encompasses supercooling, partial freezing, liquidus tracking, and isochoric subcooling, providing extended preservation times and reduced ice formation, while remaining technically complex, subject to metastability, and limitedly validated clinically. Deep-subzero preservation (−80 °C to −196 °C) includes conventional cryopreservation, vitrification, isochoric vitrification, and nanowarming, enabling long-term storage and centralized tissue banking at the cost of ice-related injury, CPA toxicity, and osmotic stress, as well as warming-associated damage.

The schematic overview of the aforementioned methods, along with their advantages and drawbacks, is presented in Figure 1.

## Cryopreservation of human vascular tissues

### Background

The need for human vascular tissue in surgical practice, as grafts, conduits or patches, cannot be overstated. The availability of banked blood vessels is crucial especially when prosthetic or tissue-engineered substitutes are not efficient and destined to fail. There is also a substantial demand for vascular tissue in physiological, pharmacological and clinical research related to angiology and cardiology. Currently, isolated vascular tissue specimens can be sourced as explanted autogenic, allogenic, or xenogenic biological material, prosthetic implants made of synthetic polymers, or tissue-engineered vessel substitutes.

In brief, venous conduits are needed for coronary artery bypass grafting (CABG), lower limb vein bypass, carotid or femoral/popliteal endarterectomy, arteriovenous fistula, or portal vein reconstruction. Arterial conduits are needed for peripheral artery diseases, CABG, and traumatic injuries. Vascular grafts in general are needed for limb salvage in critical ischemia, infection associated with previous grafting, and congenital malformations. Considering the prevalence of such conditions, the huge demand for vascular tissue appears quite warranted, while also justifying the crucial role of the temperature-dependent preservation of specimens, harvested either for surgery or for research.

At a first glance, although blood vessels are multicellular systems, their cryopreservation may appear to be less problematic than that of full solid organs. The effects of cryopreservation on the structure, functionality and properties of excised human vascular tissue have been extensively investigated since the early 1980s by Müller-Schweinitzer and colleagues at Sandoz Ltd. and at University of Basel (Müller-Schweinitzer et al., 1986; Elis and Müller-Schweinitzer, 1991; Müller-Schweinitzer, 1992; Müller-Schweinitzer et al., 1994; Müller-Schweinitzer et al., 1997; Müller-Schweinitzer et al., 1998; Müller-Schweinitzer et al., 2000; Müller-S et al., 2005; Müller-Schweinitzer et al., 2007); to our knowledge, Müller-Schweinitzer also has published the only review available to date on the subject (Müller-Schweinitzer, 2009). These investigations addressed both arterial and venous segments, such as aortas, iliac, pulmonary and mammary arteries, and saphenous veins. As expected, some results were obtained on experimental animals, and generally confirmed in human specimens. In this article, we focus on the latter, and summarize below Müller-Schweinitzer’s findings.

The main parameters investigated post-cryopreservation, which ideally must be maintained unchanged or be little affected during freezing/thawing (respectively cooling/warming) of the excised vascular tissue included:

- Smooth muscle function (e.g., contractile response and calcium uptake)
- Activity of monoamine oxidase
- Endogenous prostaglandin synthesis
- Endothelial function
- Adrenergic neurotransmission
- Elastic properties

As for other parameters, it was noticed that the levels of tissular enzymes, such as lactic dehydrogenase, glutamic transaminases, or creatine phosphokinase, increased in human vessels following cryopreservation, albeit its significance was not fully understood. Also observed was the reduction of compliance after the stored specimens were transplanted and reversed to normal hemodynamic conditions. In addition, it was concluded that densely packed cells (like in arteries) are more susceptible to cryodamage; therefore, the veins, where cells are loosely packed, appear less prone to cryodamage.

The post-cryopreservation stability of the above criteria, especially of the endothelial function, a foremost regulator of vascular homeostasis, is essential for the long-term viability of vascular grafts. In principle, the main factors that determine the occurrence of cryodamage include the rates of freezing/thawing (which are responsible for unwanted thermal stress), the stepwise techniques applied to attain the optimum temperature conditions, and the nature of cryomedium as well the procedures for its addition and removal (which can cause osmotic shock). A successful outcome in using the cryopreserved human vascular tissue for surgery or research without controlling or attempting to control the above factors is perhaps unachievable. It is important to note that the mentioned studies largely concluded that the deep-subzero cryopreservation had generally minimum negative effects on the functional criteria of the stored vascular tissue.

Nevertheless, findings partially disagreeing with the above conclusions have been reported. For instance, in human descending aortic segments cryopreserved at −197 °C for 6 weeks, the SMC contractility was “strongly affected”, although other functions were maintained (Langerak et al., 2001). In another study (Pasquinelli et al., 2006), the storage of human allogenic thoracic aortic segments at −120 °C in DMSO has caused damage to SMC function. At the other end of the scale, viable human mesenchymal stromal/stem cells were successfully isolated from human arteries that were stored in liquid nitrogen for 5 years (Valente et al., 2014), a significant proof for the validity of deep-subzero cryopreservation, both as a conservation method and as a tool for the on-demand procurement of stem cells for patients in need of cell therapies.

In vascular and cardiovascular surgery, cryopreserved arterial allografts (CAAs) offer significant advantages for managing vascular graft and endograft infections, particularly when *in situ* reconstruction is required in contaminated or infected regions (Couture et al., 2021; Weiss et al., 2021; Ben et al., 2018; Janko et al., 2019; Antonopoulos et al., 2019; Heinola et al., 2019; Tabiei et al., 2024; Lejay et al., 2017; Furlough et al., 2019; Mestres et al., 2019). Cryopreserved grafts consistently show high resistance to reinfection compared to prosthetic materials, thanks to their biological makeup, preserved extracellular matrix, and lower susceptibility to bacterial colonization, even amid polymicrobial or enteric contamination. Extensive clinical studies and long-term observations report acceptable reinfection rates of 10%–12%, which are favorable relative to rifampicin-soaked or silver-coated prostheses, particularly in cases involving aortoenteric fistula or aggressive pathogens (Couture et al., 2021; Weiss et al., 2021; Ben et al., 2018).

Another significant advantage of cryopreserved allografts is their immediate availability, eliminating the need for time-intensive autologous vein harvesting. This reduction in operative duration subsequently alleviates physiological stress in critically ill or septic patients and facilitates their use in emergency scenarios (Janko et al., 2019). From a mechanical perspective, CAAs offer a robust, thick-walled, and anatomically compatible conduit, well-suited to withstand systemic arterial pressures, especially in aortic and suprainguinal reconstructions, and demonstrate satisfactory early- and mid-term patency and limb-salvage rates (Couture et al., 2021; Antonopoulos et al., 2019; Heinola et al., 2019). Meta-analyses and multicenter studies further confirm their durability, showing acceptable 30-day mortality rates, low allograft-related mortality during follow-up, and a reasonable rate of major graft-related complications. This supports their use as a viable alternative to extra-anatomic bypass or autologous vein reconstruction when those options are unavailable or contraindicated (Antonopoulos et al., 2019). Although late complications such as stenosis, thrombosis, pseudoaneurysm formation, or graft degeneration may occur and necessitate close imaging surveillance, these events are often manageable with secondary interventions and are generally associated with low rates of amputation and graft-related death (Tabiei et al., 2024; Lejay et al., 2017; Furlough et al., 2019; Mestres et al., 2019). Overall, the accumulated evidence supports cryopreserved grafts as an infection-resistant, anatomically suitable, and clinically effective option for complex vascular graft and endograft infections, particularly in high-risk patients where durable *in situ* reconstruction is essential.

### Overview

The literature related to post-cryopreservation studies on the animal vascular tissues is relatively vast, and it has its contribution with valuable information to the research on human tissue, although the two directions of research became virtually contemporaneous in the 19th century. While our aim is to review here the research on human tissues only, we shall however mention here a brief selection of significant reports published on the animal vascular tissue of various species, such as rabbit (Hunt et al., 1994; Pegg et al., 1997; Bateson et al., 1994; Song et al., 1994; Song et al., 1995; Song et al., 2000a; Cui et al., 2002; Zhang et al., 2005; Baicu et al., 2006; Baicu et al., 2008), dog (Pierce et al., 1949; Dent et al., 1974; Weber et al., 1975a; Weber et al., 1975b; Brockbank et al., 1994), pig (Medynsky et al., 1998; Pascual et al., 2001; Venkatasubramanian et al., 2006; Stemper et al., 2007; Wille et al., 2008; O’Leary et al., 2014; He et al., 2022), rat (Ingemansson et al., 1996; Gabriel et al., 2006; Zatschler et al., 2009; Ren et al., 2023a), bovine (Chow and Zhang, 2011), or ovine (Smardencas and Birchall, 2011), which might have furthered advancements in the field.

The literature involving the preservation of human vascular tissues is also considerable in volume, and to cover it toward completion is associated with hard labor and limited utility. Here, we summarize representative reports that led to the current level of knowledge and applications in the field. The studies using frozen vascular tissue purchased from commercial suppliers, or applications reported without details of the preservation method, have not been included in our review.

*Hypothermic method* was frequently employed during the early period of modern vascular surgery, and Carrel’s work has been mentioned in a previous section. Notable work was carried out by Gross and colleagues at Harvard, using an ice box or a refrigerator to achieve temperatures between 1 °C and 4 °C (Gross et al., 1948; Gross et al., 1949). Human arterial segments (iliac, carotid, subclavian, aortic) were stored at these temperatures for up to 40 days, in a solution containing glucose, human serum, bicarbonate buffer, penicillin and streptomycin. They were implanted as shunts for bridging gaps between aortic system and pulmonary artery, or for correction of coarctation of thoracic aorta. Although the follow-up stage was short, it was concluded that the results were promising, but no correlation with the storage method was discussed.

In time, the compositions of the media for hypothermic storage became more intricate. For instance, successful hepatic aneurysmectomy and revascularization was achieved using iliac arterial allografts that were preserved for up to 30 days at 4 °C–10 °C in RPMI 1640 medium mixed with a palette of four antibiotics (Sellers et al., 2002). The RPMI 1640 was initially developed as a cell culture medium, and contains glucose, salts of sodium, potassium, magnesium, and calcium, amino acids, vitamins, and pH indicators. Biomechanical properties of the grafts preserved in hypothermic conditions have been used as a criterion for comparing their performance with those of cryopreserved grafts. Thus, the measured mechanical parameters (modulus, stress) of human descending aortic allografts stored up to 31 days at 4 °C in a medium consisting of EuroCollins solution mixed with antibiotics showed no significant difference when compared with grafts stored at −135 °C (vapor phase liquid nitrogen) in DMSO for up to 4 months (Adham et al., 1996). Perhaps the same storage duration would have been more relevant for a conclusion. EuroCollins solution was developed as a preservation medium for the hypothermic preservation of solid organs, and is composed of glucose and salts (phosphate, bicarbonate, chloride) of sodium and/or potassium. A study (Garbe et al., 2011) comparing four different solutions used for the preservation at 4 °C of human internal mammary arterial segments, collected from a large number of patients, has been carried out aiming at finding the most suitable medium. The media tested included TiProtec (newly developed in Germany at that time), histidine-tryptophan-ketoglutarate, phosphate-buffered saline (PBS), and sodium chloride 0.9% solutions. The specimens were assessed post-rewarming for the maintenance of specific functions, including vessel tone, smooth muscle relaxation, endothelial relaxation, and tissue reductive capacity. While those parameters became impaired within days in most media, the storage in TiProtec assured a prolonged cold storage alternative, up to 25 days (Garbe et al., 2011). This rather sophisticated medium was specially developed for the conservation of vascular tissue, and consists of N-acetylhistidine, fortified with amino acids and carbohydrates, enriched in potassium, buffered, and containing the iron chelating agents deferoxamine and LK 614 (N-hydroxy-3,4-dimethoxy-N-methylbenzamide). TiProtec was further investigated (Buchinger-Kähler et al., 2016) as a conservation medium in the hypothermic (4 °C) preservation procedure of great saphenous venous tissue harvested from patients undergoing aorto-coronary venous bypass surgery. In that study, TiProtec was compared with both KHB solution and the University of Wisconsin solution (UWS), and the criteria were used for a thorough assessment including contractile functions, endothelial relaxation, and cellular morphology. Based on such functional outcomes, TiProtec medium was suggested as the most promising alternative for vein cold storage. UWS is a complex medium that was developed for conservation of kidneys, liver, or pancreas, and contains glutathione, adenosine, allopurinol, osmotic agents, hydroxyethyl starch, and potassium and sodium compounds. In a recent *in vitro* study (Ren et al., 2023b), two preservation methods (4 °C and −186 °C) were compared regarding their performance for the storage of human iliac veins, while unprocessed fresh tissue served as initial control. The medium for both methods was a mixture of antibiotics, saline, and Medium 199 (for cell culture), with the addition of DMSO for the freezing stage. Tests were carried out up to 28 days to evaluate cellular metabolic activity, wall structure, mechanical strength, and histological aspects. It was concluded that both methods were able to maintain effectively the properties of venous specimens. In another recent study (Bordet et al., 2024), human femoropopliteal arterial allografts were stored up to 12 months at temperatures between 4 °C and 8 °C in solution of saline and antibiotics, and their mechanical properties were compared to those of cryopreserved grafts. The hypothermic storage up to 12 months did not have any notable effect on biomechanics of the grafts.

Hypothermic and subnormothermic preservation of grafts are important in cardiovascular surgery, especially for performing CABG, where a relatively short period of time is permitted between procurement of graft and its insertion into the anastomosis. Some grafts, such as internal mammary artery can be left *in situ*, but most have to be flushed with, and stored in a suitable medium during that short period. Media for the intraoperative preservation of grafts and their effects on grafts’ long-term patency have been critically discussed in recent excellent reviews (Tsakok et al., 2012; Winkler et al., 2016; Woodward et al., 2016; Toto et al., 2022). Beside the classic saline and autologous whole blood, alternative formulations have been developed, aiming at minimizing the endothelial damage induced during intraoperative conservation of vascular grafts. Indeed, it was noticed some time ago (Thatte and Khuri, 2001) that endothelial damage may be a major cause of graft failure.

Certain solutions used for the hypothermic, subnormothermic and normothermic intraoperative preservation of human vascular tissues were initially produced and marketed for organ preservation. New media are in continuous development (Jing et al., 2018; Chen et al., 2019; Soo et al., 2020); quite likely, some of them will eventually be applied to preserve vascular grafts.

Marangoni and Cecchini were the first to use *lyophilization (freeze-drying)* for the preservation of arterial segments (Marangoni and Cecchini, 1951), but not of human origin. They implanted canine femoral and aortic allografts for bridging arterial defects, with good results after 3 months’ follow-up. The grafts had been lyophilized and stored at room temperature for as long as 60 days before surgery. The freezing temperature was not below −25 °C. Remarkably, the lyophilized grafts recovered their native elasticity upon re-hydration prior to use. In the 1950s, lyophilization became popular for the preservation of human arterial grafts (Brown et al., 1953; Creech et al., 1954; R and ob, 1954; Hammer et al., 1954; Fisher et al., 1956; Foster et al., 1958). We shall mention that, much earlier, Carrel preserved arterial grafts by desiccation over anhydrous calcium chloride (Carrel, 1910), such foreshadowing the drying as a preservation method and proving once more his visionary genius. First clinical application of lyophilized arterial allografts in human patients was reported by Molnar et al. (2012). The freezing temperature was −78 °C, the vacuum was 0.05–0.1 mmHg, and the reconstitution of vascular segment was achieved in saline with antibiotics. The grafts were employed to repair occlusions and aneurysms in seven patients, with mixed outcomes. In another study (Merivaara et al., 2021), lyophilized (−78 °C, 0.0005 mmHg) arterial allografts were used to repair over 50 cases of aneurysms and occlusive lesions. No complications have been attributed to the lyophilization method. In a larger trial (Foster et al., 1958), over 100 patients were surgically treated for aneurysms, occlusions, trauma, or coarctation) using freeze-dried (−76 °C) arterial grafts, with satisfactory outcomes. Prior to lyophilization, the grafts were sterilized in liquid ethylene oxide.

Application of lyophilization for preserving venous grafts has occurred later, and to a lesser extent. Various venous segments excised post-mortem from young subjects were lyophilized (−60 °C, 0.05 mmHg) and then investigated by scanning electron microscopy (Schulte-Wrede et al., 1975). The wall structures (endothelium, intima, media, and adventitia) remained virtually unchanged, and it was concluded that lyophilization could provide venous grafts suitable for surgery and able to replace successfully the arterial grafts. Lyophilization of saphenous vein allografts has also attracted interest (Merrill et al., 1979; Reeves et al., 1997; Timaran et al., 2002). For instance, arteriovenous fistulae for hemodialysis were created in 11 human patients using lyophilized human saphenous vein allografts, and followed for up to 14 months (Merrill et al., 1979). The outcomes were compared to those of commercially available bovine xenografts, and the advantages of lyophilization were acknowledged. In another study (Timaran et al., 2002), lyophilization (−60 °C, 1 mmHg, reconstitution in buffered saline) was employed to preserve human saphenous vein allografts for treating femoropopliteal occlusive disease.

In spite of its many recognized advantages, lyophilization has not become a routine preservation method of biological materials, vascular tissue included. Currently, it seems that this situation has triggered additional research, as discussed in some recent publications (Molnar et al., 2012; Merivaara et al., 2021).

The relatively newer deep-subzero technique of *vitrification* presents the advantage of avoiding ice formation, but requires large amounts of CPAs, as detailed in a previous section. Its use for vascular tissue shared the same fate like the lyophilization procedure, in that not being applied routinely as a low-temperature preservation method. There have been favorable reports on the effects and performance of rabbit veins (Song et al., 2000a; Song et al., 2000b) and tissue-engineered vessels (Dahl et al., 2006) conserved by vitrification. A detailed study (Thakrar et al., 2006) of human iliac arteries processed by vitrification, and compared with tissue processed by a traditional deep-subzero method, indicated that biomechanical characteristics were maintained in the vitrified specimens to a much higher level than those of cryopreserved specimens when both compared with fresh tissue. The cryomedium consisted of mixtures of 1,2-propanediol (as a CPA) and DMEM, and the final temperature was −196 °C (liquid nitrogen). For vitrification, the amount of CPA was 4 times higher than that used for cryopreservation. In another study (Mallis et al., 2020), human umbilical arteries were decellularized and grafted in a porcine model. The grafts specimens subjected to vitrification (in a medium consisting of DMSO, 1,2-propanediol, formamide, and EuroCollins solution) maintained structural and functional properties better than those subjected to a traditional deep-subzero method. More recently (Cao et al., 2021), the thermal effect induced by a magnetic field heating was employed to facilitate the warming stage in the vitrification of human umbilical arteries. The mechanical properties were found improved due to less thermal stress during warming stage.

*Traditional cryopreservation* techniques of vascular tissue at subzero temperatures were applied to a larger extent than any other procedures. In addition to the cases already discussed throughout our review, Table 1 presents a compilation of selected publications related to the preservation of human vascular grafts at subzero temperatures, covering four decades of reported activity. A variety of cryomedia were used, where DMSO was ubiquitous, and occasionally they included agents that were initially developed for cell culture or preservation of organs. Notably, the cryogenic conditions *per se* were not perceived as causing episodically poor clinical outcomes in any of the studies (Ochsner et al., 1984; Gelbfish et al., 1986; Brockbank, 1994; Rosset et al., 1996; Lesèche et al., 1997; Castier et al., 1999; Pukacki et al., 2000; Armentano et al., 2006; Santana et al., 2007; Bisdas et al., 2010; Aydin et al., 2013; Touma et al., 2014; Ha et al., 2016; Jashari et al., 2023) cited in Table 1.

**TABLE 1:** Overview of selected literature on the storage of human vascular tissue at subzero temperatures.

Type of allograft tissue	Method of cryopreservation	Cryomedium	Storage duration	Surgical or investigational use	Source
Saphenous vein	−70 °C	Antibiotic solution	n.a.	Limb salvage	Ochsner et al. (1984)
Mammary artery	−50 °C	No medium	up to 118 days	Coronary artery bypass	Gelbfish et al. (1986)
Saphenous vein	<–135 °C	DMSO Chondroitin sulfate DMEM	up to 2 years	Research: Functional analysis *in vitro*	Brockbank (1994)
Common carotid artery Superficial femoral artery	−140 °C^a^	DMSO	4.2 months (average)	Research: Mechanical evaluation	Rosset et al. (1996)
Saphenous vein	−80 °C	DMSO	n.a.	Limb salvage	Lesèche et al. (1997)
Artery	−80 °C	DMSO M199 medium^b^	83 days (average)	Limb salvage	Castier et al. (1999)
Artery Vein	−80 °C^c^	DMSO	2–4 weeks	Research: Mechanical evaluation	Pukacki et al. (2000)
Common carotid artery	−90 °C; −142 °C^a^	DMSO RPMI 1640 medium^d^ Human albumin	30 days	Research: Mechanical evaluation	Armentano et al. (2006)
Femoral artery	As above	As above	n.a.	Research: Functional analysis *in vitro*	Santana et al. (2007)
Artery	−40 °C; −100 °C^c^	DMSO M199 medium^b^	n.a.	Reconstruction of infected aorta	Bisdas et al. (2010)
Iliac artery Abdominal aorta Vein (saphenous, cava)	−22 °C	Ringer lactate^e^	up to 3 months	Vascular reconstruction during liver transplantation	Aydin et al. (2013)
Artery	(i) −50 °C; −140 °C^a^ (ii) −80 °C	(i) DMSO Human albumin (ii) DMSO SCOT solution^f^	up to 2 years	Aortic infections	Touma et al. (2014)
Artery	−70 °C; −196 °C^g^	DMSO	n.a.	Arteriovenous fistula for hemodialysis	Ha et al. (2016)
Aorta Iliac artery Femoral artery Pulmonary artery	−178 °C^a^	DMSO RPMI 1640 medium^d^	n.a.	Limb salvage Infected grafts Infected aneurysms Congenital cardiac disease	Jashari et al. (2023)

Abbreviations: DMSO, dimethyl sulfoxide; DMEM, Dulbecco’s modified Eagle’s medium; ECM, extracellular matrix; n.a., information not available.

^a^ Liquid nitrogen vapour phase (no immersion).

^b^ Cell culture medium containing glucose, amino acids, vitamins, cholesterol, pyrimidines etc.

^c^ Followed by exposure to nitrogen vapour phase.

^d^ Cell culture medium containing glucose, amino acids, vitamins, salts, pH indicators etc.

^e^ Solution with an osmolarity lower than saline, containing sodium lactate and other salts.

^f^ Medium for renal transplantation containing ECM, ionic components and poly(ethylene glycol) (20 kDa). Not to be confused with SCOTT, solution.

^g^ Immersion in liquid nitrogen.

## New directions in biopreservation

Recent advances in biopreservation are increasingly focused on extending storage duration while preserving vascular structure and endothelial function. Traditional hypothermic storage at 4 °C remains constrained by ischemic injury and progressive endothelial dysfunction, prompting the exploration of alternative temperature ranges and biologically targeted preservation strategies (Bordet et al., 2024; Hwang et al., 2021). High-subzero preservation approaches, including isochoric preservation, represent a promising direction by enabling lower-temperature storage while suppressing ice formation through constant-volume thermodynamics (Rubinsk et al., 2005; Năstase et al., 2023). These methods reduce mechanical and osmotic stress and may be particularly advantageous for vascular tissues, which are highly susceptible to ice-induced damage. In parallel, progress in vitrification has highlighted the critical importance of rapid and uniform rewarming; volumetric heating techniques such as nanowarming have been developed to mitigate thermal gradients and structural injury during warming (Manuchehrab et al., 2017). Beyond thermal control, new preservation solutions and transport systems are being designed to support multi-day storage while reducing ischemic and reperfusion-associated injury. These formulations increasingly emphasize endothelial protection, oxidative stress reduction, and metabolic suppression rather than osmotic balance alone. Technologies such as XT-ViVo® and TimeSeal® exemplify this trend and may help streamline vascular tissue logistics as validation data continue to accumulate (Heberle et al., 2025; Dong et al., 2025; Hassan et al., 2025; Khaki et al., 2025; Muss et al., 2025; Kim et al., 2025). However, despite preliminary data presented at kidney transplant conferences, no data have been published in the current literature to date (Heberle et al., 2025; Dong et al., 2025; Hassan et al., 2025; Khaki et al., 2025; Muss et al., 2025; Kim et al., 2025). Collectively, these emerging strategies signal a shift toward vascular biopreservation paradigms that integrate optimized temperature management with functional endothelial preservation, supporting longer storage times without compromising post-transplant performance.

## Conclusion

A large variety of preservation procedures, ranging from normothermic to deep-subzero temperatures, have been used for the storage of human vascular tissue intended for surgery or research. The procedures present different and specific advantages or disadvantages when compared to each other. Normothermic and hypothermic preservation procedures reduce substantially the costs, but are of limited use and only for short durations. Despite certain advantages (e.g., it avoids the use of liquid nitrogen, assures long duration storage without supervision, and reduces storage and shipping costs), lyophilization could not find a steady niche in the preservation of vascular grafts. Vitrification appears to be effective in reducing substantially the cryodamage, but currently is not frequently applied, one of the likely reasons being the costs involved. The traditional subzero-temperature procedures seem to be the preferred alternative for storing vascular tissue, in spite of obvious disadvantages such as need for liquid nitrogen, and high costs for equipment and shipping. If performed following strictly established protocols, and if associated with immunological monitoring, the conventional approaches to subzero cryopreservation methodology may reduce cryodamage to safe levels and can increase duration of storage, such contributing to successful clinical outcomes.

Whereas the majority of topical publications have reported favorable results irrespective of the preservation procedure, to recommend a particular technique is rather irrelevant.

## References

[B1] Adham M. Gournier J.-P. Favre J.-P. De La Roche E. Ducerf C. Baulieux J. (1996). Mechanical characteristics of fresh and frozen human descending thoracic aorta. J. Surg. Res. 64, 32–34. 10.1006/jsre.1996.0302 8806470

[B2] Antonopoulos C. N. Papakonstantinou N. A. Hardy D. Lyden S. P. (2019). Editor's choice - cryopreserved allografts for arterial reconstruction after aorto-iliac infection: a systematic review and meta-analysis. Eur. J. Vasc. Endovasc. Surg. 58 (1), 120–128. 10.1016/j.ejvs.2019.03.003 31202580

[B3] Arav A. Natan Y. (2024). Narrative review on tissue and organ cryopreservation research related to regenerative medicine: from early attempts to future possibilities. Regen. Med. Rep. 1, 137–148. 10.4103/regenmed.regenmed-d-24-00017

[B4] Armentano R. L. Santana D. B. Cabrera Fischer E. I. Graf S. Pérez Cámpos H. Zocalo Germán Y. (2006). An *in vitro* study of cryopreserved and fresh human arteries: a comparison with ePTFE prostheses and human arteries studied non-invasively *in vivo* . Cryobiology 52, 17–26. 10.1016/j.cryobiol.2005.09.001 16274686

[B5] Asghar W. El Assal R. Shafiee H. Anchan R. M. Demirci U. (2014). Preserving human cells for regenerative, reproductive, and transfusion medicine. Biotechnol. J. 9, 895–903. 10.1002/biot.201300074 24995723 PMC4145864

[B6] Atta H. M. (1999). Edwin Smith surgical Papyrus: the oldest known surgical treatise. Am. Surg. 65, 1190–1192. 10.1177/000313489906501222 10597074

[B7] Aydin C. Ince V. Otan E. Akbulut S. Koc C. Kayaalp C. (2013). Storage of allogenic vascular grafts: experience from a high-volume liver transplant institute. Int. Surg. 98, 170–174. 10.9738/INTSURG-D-12-00035.1 23701155 PMC3723171

[B8] Baicu S. Taylor M. J. Chen Z. Rabin Y. (2006). Vitrification of carotid artery segments: an integrated study of thermophysical events and functional recovery toward scale-up for clinical applications. Cell Preserv Technol. 4, 236–244. 10.1089/cpt.2006.9994 18185850 PMC2180387

[B9] Baicu S. Taylor M. J. Chen Z. Rabin Y. (2008). Cryopreservation of carotid artery segments via vitrification subject to marginal thermal conditions: correlation of freezing visualization with functional recovery. Cryobiology 57, 1–8. 10.1016/j.cryobiol.2008.03.002 18490009 PMC3384691

[B10] Baker H. (1764). Employment for the microscope in two parts. 2nd Ed. London: R. & J. Dodsley, 250–260.

[B11] Bateson E. A. J. Busza A. L. Pegg D. E. Taylor M. J. (1994). Permeation of rabbit common carotid arteries with dimethyl sulfoxide. Cryobiology 31, 393–397. 10.1006/cryo.1994.1047 7924396

[B12] Ben A. S. Louvancourt A. Daniel G. Combe P. Duprey A. Albertini J. N. (2018). Cryopreserved arterial allografts for *in situ* reconstruction of abdominal aortic native or secondary graft infection. J. Vasc. Surg. 67 (2), 468–477. 10.1016/j.jvs.2017.06.088 28826728

[B13] Bennett H. (2025). Life on ice. Chem. World 22 (1), 34–37.

[B14] Bert P. De la Greffe A. (1863). Thèse pour le Doctorat en Médicine. Paris: Imprimerie E. Martinet, 81–82.

[B15] Bert P. De la Vitalité Propre des Tissus A. (1866). Thèse de Docteur ès Sciences Naturelles. Paris: Imprimerie E. Martinet.

[B16] Best B. P. (2015). Cryoprotectant toxicity: facts, issues, and questions. Rejuv Res. 18, 422–436. 10.1089/rej.2014.1656 25826677 PMC4620521

[B17] Biot É. (1851). Le Tcheou-Li ou Rites des Tcheou, vol. 1. Paris: Imprimerie Nationale.

[B18] Bisdas T. Bredt M. Pichlmaier M. Aper T. Wilhelmi M. Bisdas S. (2010). Eight-year experience with cryopreserved arterial homografts for the *in situ* reconstruction of abdominal aortic infections. J. Vasc. Surg. 52, 323–330. 10.1016/j.jvs.2010.02.277 20570473

[B19] Bojic S. Murray A. Bentley B. L. Spindler R. Pawlik P. Cordeiro J. L. (2021). Winter is coming: the future of cryopreservation. BMC Biol. 19, 56. 10.1186/s12915-021-00976-8 33761937 PMC7989039

[B20] Bordet M. Rival G. Seveyrat L. Millon A. Capsal J.-F. Cottinet P.-J. (2024). Cold storage of human femoral arteries for twelve months: impact on mechanical properties. Eur. J. Vasc. Endovasc. Surg. 68, 797–802. 10.1016/j.ejvs.2024.07.040 39111534

[B21] Boyle R. (1665). New experiments and observations touching cold, or an experimental history of cold. London: John Crook, 454–462.

[B22] Breasted J. H. (1930). The Edwin Smith surgical papyrus, Vol. 1. (Chicago (IL): University of Chicago Press).

[B23] Brockbank K. G. M. (1994). Effects of cryopreservation upon vein function *in vivo* . Cryobiology 31, 71–81. 10.1006/cryo.1994.1009 8156802

[B24] Brockbank K. G. M. Donovan T. J. Ruby S. T. Carpenter J. F. Hagen P.-O. Woodley M. A. (1994). Functional analysis of cryopreserved veins. J. Vasc. Surg. 11, 94–102. 10.1016/0741-5214(90)90333-6 2296107

[B25] Brown R. B. Hufnagel C. A. Pate J. W. Strong W. R. (1953). Freeze-dried arterial homografts. Surg. Gynecol. Obstet. 97, 657–664. 13113548

[B26] Buchinger-Kähler V. Stoldt V. R. Muth T. Schipke J. D. (2016). Function and viability of vessels in different preservation solutions – an experimental study on human great saphenous veins. HSOA J. Angiol. Vasc. Surg. 1, 003. 10.24966/AVS-7397/100003

[B27] Bwanga C. O. (1991). Cryopreservation of boar semen I: a literature review. Acta Vet. Scand. 32, 431–453. 10.1186/BF03546944 1818503 PMC8127938

[B28] Cao M. Xu Y. Dong Y. (2021). Improving mechanical properties of vitrified umbilical arteries with magnetic warming. Fluid Dyn. Mater Proc. 17, 123–139. 10.32604/fdmp.2021.011443

[B29] Carrel A. (1907). Heterotransplantation of blood vessels preserved in cold storage. J. Exp. Med. 9, 226–228. 10.1084/jem.9.2.226 19867084 PMC2124659

[B30] Carrel A. (1908). Results of the transplantation of blood vessels, organs and limbs. J. Am. Med. Assoc. 51, 1662–1667. 10.1001/jama.1908.25410200010001b

[B31] Carrel A. (1910). Latent life of arteries. J. Exp. Med. 12, 460–486. 10.1084/jem.12.4.460 19867337 PMC2124808

[B32] Carrel A. (1912a). Ultimate results of aortic transplantations. J. Exp. Med. 15, 389–392. 10.1084/jem.15.4.389 19867531 PMC2124929

[B33] Carrel A. (1912b). The preservation of tissue and its applications in surgery. J. Am. Med. Assoc. 59, 523–527.

[B34] Carrel A. Lindbergh C. A. (1935). The culture of whole organs. Science 81, 621–623. 10.1126/science.81.2112.621 17733174

[B35] Castier Y. Lesèche G. Palombi T. Petit M.-D. Cerceau O. (1999). Early experience with cryopreserved arterial allografts in below-knee revascularization for limb salvage. Am. J. Surg. 177, 197–202. 10.1016/s0002-9610(99)00010-0 10219854

[B36] Chang T. Zhao G. (2021). Ice inhibition for cryopreservation: materials, strategies, and challenges. Adv. Sci. 8, 2002425. 10.1002/advs.202002425 33747720 PMC7967093

[B37] Chen Y. Shi J. Xia T. C. Xu R. He X. Xia Y. (2019). Preservation solutions for kidney transplantation: history, advances and mechanisms. Cell Transpl. 28, 1472–1489. 10.1177/0963689719872699 31450971 PMC6923544

[B38] Chen J. Liu X. Hu Y. Chen X. Tan S. (2023). Cryopreservation of tissues and organs: present, bottlenecks, and future. Front. Vet. Sci. 10, 1201794. 10.3389/fvets.2023.1201794 37303729 PMC10248239

[B39] Chow M.-J. Zhang Y. (2011). Changes in the mechanical and biochemical properties of aortic tissue due to cold storage. J. Surg. Res. 171, 434–442. 10.1016/j.jss.2010.04.007 20701927

[B40] Clark A. (1898). ‘Brief lives’, chiefly of contemporaries, set Down by John Aubrey, between the years 1669 & 1696, Vol. 1. (Oxford: Clarendon Press), 75–76.

[B41] Coriell L. L. Greene A. E. Silver R. K. (1964). Historical development of cell and tissue culture freezing. Cryobiology 1, 72–79. 10.1016/0011-2240(64)90024-0 14247546

[B42] Couture T. Gaudric J. Du Montcel S. T. Jayet J. Verscheure D. Davaine J. M. (2021). Short and mid term outcomes of cryopreserved abdominal aortic allografts used as a substitute for infected prosthetic grafts in 200 patients. Eur. J. Vasc. Endovasc. Surg. 62 (1), 89–97. 10.1016/j.ejvs.2021.02.036 33858752

[B43] Creech Jr O. De Bakey M. E. Cooley D. A. Self M. M. (1954). Preparation and use of freeze-dried arterial homografts. Ann. Surg. 140, 35–43. 10.1097/00000658-195407000-00003 13159141 PMC1609601

[B44] Criswell T. Swart C. Stoudemire J. Brockbank K. G. M. Powell-Palm M. Stilwell R. (2023). Freezing biological time: a modern perspective on organ preservation. Stem Cells Transl. Med. 12, 1–6. 10.1093/stcltm/szac083 36571240 PMC9887086

[B45] Cui X. Labarrere C. He L. Cheng S. Siderys H. Kovacs R. (2002). Cryopreservation and microsurgical implantation of rabbit carotid artery. Cell Preserv Technol. 1, 121–128. 10.1089/153834402320882629

[B46] Dahl S. L. M. Chen Z. Solan A. K. Brockbank K. G. M. Niklason L. E. Song Y. C. (2006). Feasibility of vitrification as a storage method for tissue-engineered blood vessels. Tissue Eng. 12, 291–300. 10.1089/ten.2006.12.291 16548687

[B47] Dent T. L. Weber T. R. Lindenauer S. M. Ascher N. Weatherbee L. Allen E. (1974). Cryopreservation of vein grafts. Surg. Forum 25, 241–243. 4439177

[B48] Dong Y. Chen M. Ju W. He X. (2025). Normothermic machine perfusion of porcine small intestines from donation after cardiac death. AJT 25 (8), S795. 10.1016/j.ajt.2025.07.1878

[B49] Elis P. Müller-Schweinitzer E. (1991). Maintenance of functional activity of human pulmonary arteries after cryopreservation. Br. J. Pharmacol. 103, 1377–1380. 10.1111/j.1476-5381.1991.tb09797.x 1884097 PMC1908380

[B50] Elliott G. D. Wang S. Fuller B. J. (2017). Cryoprotectants: a review of the actions and applications of cryoprotective solutes that modulate cell recovery from ultra-low temperatures. Cryobiology 76, 74–91. 10.1016/j.cryobiol.2017.04.004 28428046

[B51] Ellison E. R. (1978). A study of diet in Mesopotamia (c. 3000-600 BC) and associated agricultural techniques and methods of food preparation. London: University of London, 154. Available online at: http://discovery.ucl.ac.uk/id/eprint/1349279/1/454702_vol1.pdf (Accessed January 30, 2025).

[B52] Eskandari A. Leow T. C. Rahman M. B. A. Oslan S. N. (2020). Antifreeze proteins and their practical utilization in industry, medicine, and agriculture. Biomolecules 10, 1649. 10.3390/biom10121649 33317024 PMC7764015

[B53] Fahy G. M. Wowk B. (2015). “Principles of cryopreservation by vitrification,” in Cryopreservation and freeze-drying protocols. (New York: Springer Science Business Media), 21–82. 10.1007/978-1-4939-2193-5_2 25428002

[B54] Feldman R. P. Goodrich J. T. (1999). The Edwin Smith surgical papyrus. Child’s Nerv. Syst. 15, 281–284. 10.1007/s003810050395 10461775

[B55] Fisher B. Adams C. Wilde R. Fisher E. R. Dattilo J. (1956). The sterilization and storage of lyophilized blood vessels. Ann. Surg. 143, 73–80. 10.1097/00000658-195601000-00009 13275901 PMC1464945

[B56] Foster J. H. Lance E. M. Scott H. W. (1958). Experience with ethylene oxide treated freeze-dry arterial homografts in 110 consecutive patients. Ann. Surg. 148, 230–238. 10.1097/00000658-195808000-00012 13571899 PMC1450786

[B57] Francis Bacon D. B. C. (1993). The temper of a man. New York: Fordham University Press, 224–225.

[B58] Freitas-Ribeiro S. Reis R. L. Pirraco R. P. (2022). Long-term and short-term preservation strategies for tissue engineering and regenerative medicine products: state of the art and emerging trends. PNAS Nexus 1, 1–15. 10.1093/pnasnexus/pgac212 36714838 PMC9802477

[B59] Fu F. Dang W. He X. Xu F. Huang H. (2022). Biomolecular pathways of cryoinjuries in low-temperature storage for mammalian specimen. Bioengineering 9, 545. 10.3390/bioengineering9100545 36290513 PMC9598205

[B60] Furlough C. L. Jain A. K. Ho K. J. Rodriguez H. E. Tomita T. M. Eskandari M. K. (2019). Peripheral artery reconstructions using cryopreserved arterial allografts in infected fields. J. Vasc. Surg. 70 (2), 562–568. 10.1016/j.jvs.2018.10.111 30737000

[B61] Gabriel M. Wachal K. Dzieciuchowicz L. Pawlaczyk K. Krasinski Z. Oszkinis G. (2006). The influence of cryopreservation on changes in diameter and compliance of allografts in an animal experimental model. Eur. J. Vasc. Endovasc. Surg. 32, 169–175. 10.1016/j.ejvs.2006.01.025 16564709

[B62] Garbe S. Zatschler B. Müller B. Dieterich P. Ebner A. Rauen U. (2011). Preservation of human artery function following prolonged cold storage with a new solution. J. Vasc. Surg. 53, 1063–1070. 10.1016/j.jvs.2010.10.093 21227623

[B63] Gelbfish J. Jacobowitz I. J. Rose D. M. Connolly M. W. Acinapura A. J. Zisbrod Z. (1986). Cryopreserved homologous saphenous vein: early and late patency in coronary artery bypass surgical procedures. Ann. Thorac. Surg. 42, 70–73. 10.1016/s0003-4975(10)61839-5 3488041

[B64] Gross R. E. Hurwitt E. S. Bill Jr A. H. Peirce E. C. I. I. (1948). Preliminary observations on the use of human arterial grafts in the treatment of certain cardiovascular defects. New Engl. J. Med. 239, 578–579. 10.1056/nejm194810142391604 18886966

[B65] Gross R. E. Bill A. H. Peirce E. C. (1949). Methods for preservation and transplantation of arterial grafts. Surg. Gynecol. Obstet. 88, 689–701. 18130309

[B66] Ha T.-Y. Kim Y. H. Chang J. W. Park Y. Han Y. Kwon H. (2016). Clinical outcomes of cryopreserved arterial allograft used as a vascular conduit for hemodialysis. J. Korean Med. Sci. 31, 1266–1272. 10.3346/jkms.2016.31.8.1266 27478338 PMC4951557

[B67] Hammer J. M. Seay P. H. De Groat A. Prust F. W. (1954). Preserving arterial segments for the blood vessel bank. Arch. Surg. 69, 97–100. 10.1001/archsurg.1954.01270010099015 13170930

[B68] Hassan M. Taggart M. Lyon A. Taveras C. Ozgur O. Cutrone A. (2025). Long term enhanced subnormothermic machine perfusion of discarded human and porcine kidney grafts. AJT 25 (8), S795. 10.1016/j.ajt.2025.07.1879

[B69] He B. Musk G. C. Ng Z. Q. Kershaw H. DeBoer B. Hamdorf J. M. (2022). Investigation of a method for long-term preservation of the vascular allograft. Vascular 30, 568–576. 10.1177/17085381211012945 33966508

[B70] Heberle A. Loftin A. Pühringer-Sturmayr M. Hofmann J. Hautz T. Schneeberger S. (2025). First series of successful subzero kidney transports aboard a commercial transatlantic flight. AJT 25 (Issue 8), S795–S796. 10.1016/j.ajt.2025.07.1880

[B71] Heinola I. Kantonen I. Mattila I. Albäck A. Venermo M. (2019). Cryopreserved venous allografts in supra-inguinal reconstructions: a single centre experience. Eur. J. Vasc. Endovasc. Surg. 58 (6), 912–919. 10.1016/j.ejvs.2019.06.024 31631006

[B72] Hoole S. (1807). The select works of antony van Leeuwenhoek, containing his microscopical discoveries in many of the works of nature, vol. 2, part 3. (London: Philanthropic Society), 207–214.

[B73] Hunt C. J. Song Y. C. Bateson E. A. J. Pegg D. E. (1994). Fractures in cryopreserved arteries. Cryobiology 31, 506–515. 10.1006/cryo.1994.1061 7988160

[B74] Hwang S. Bae J. H. Kim I. O. Hong J. J. (2021). Current vascular allograft procurement, cryopreservation and transplantation techniques in the Asan medical center tissue bank. Ann. Liver Transpl. 1 (1), 79–85. 10.52604/alt.21.0016

[B75] Ingemansson R. Budrikis A. Bolys R. Sjöberg T. Steen S. (1996). Effect of temperature in long-term preservation of vascular endothelial and smooth muscle function. Ann. Thorac. Surg. 61, 1413–1417. 10.1016/0003-4975(96)00109-9 8633951

[B76] Janko M. R. Bose S. Lawrence P. F. (2019). Current status of treatment for aortic graft infection: when should cryopreserved allografts be used? Semin. Vasc. Surg. 32 (1-2), 81–87. 10.1053/j.semvascsurg.2019.07.001 31540661

[B77] Jardine L. Stewart A. (1998). Hostage to fortune: the troubled life of francis bacon. New York: Hill & Wang, 502–511.

[B78] Jashari R. Bouzet V. Alcaraz Blanco M.-J. Oleffe A. Lecocq E. Mastrobuoni S. (2023). Vascular allografts for clinical application in Europe: assessment of 30 years of experience with vascular tissue banking in Brussels. Cell Tissue Bank. 24, 613–625. 10.1007/s10561-022-10063-z 36595150 PMC9809507

[B79] Jing L. Yao L. Zhao M. Peng L. Liu M. (2018). Organ preservation: from the past to the future. Acta Pharmacol. Sin. 39, 845–857. 10.1038/aps.2017.182 29565040 PMC5943901

[B80] Karlsson J. O. M. Toner M. (1996). Long-term storage of tissues by cryopreservation: critical issues. Biomaterials 17, 243–256. 10.1016/0142-9612(96)85562-1 8745321

[B81] Keilin D. (1959). The problem of anabiosis or latent life: history and current concept. Proc. R. Soc. Lond. Ser. B 150, 149–191. 10.1098/rspb.1959.0013 13633975

[B82] KeshishianHarrison’s H. R. (2004). The outgrowth of the nerve fiber as a mode of protoplasmic movement. J. Exp. Zool. 301A, 201–203. 10.1002/jez.a.20032 14981777

[B83] Khaki S. Guo Y. Kim H. Zhang Y. Wei X. Kline M. (2025). Extended subzero organ preservation with novel bio-inspired cryoprotectants: *in vivo* assessment in a mouse heart transplantation model. AJT 25 (Issue 8), S563–S564. 10.1016/j.ajt.2025.07.1315

[B84] Khaydukova I. V. Ivannikova V. M. Zhidkov D. A. Belikov N. V. Peshkova M. A. Timashev P. S. (2024). Current state and challenges of tissue and organ cryopreservation in biobanking. Int. J. Mol. Sci. 25, 11124. 10.3390/ijms252011124 39456905 PMC11508709

[B85] Kim H. Loftin A. Wei X. Kline M. Brandacher G. Oh B. (2025). Subzero preservation minimizes histopathological damage in primary kidney cells relative to static cold storage. AJT 25 (8), S571–S572. 10.1016/j.ajt.2025.07.1335

[B86] Landecker H. (2024). Cell freezing and the biology of inexorability: on cryoprotectants and chemical time. BioSocieties 19, 635–655. 10.1057/s41292-024-00331-4 39552728 PMC11564080

[B87] Langerak S. E. Groenink M. van der Wall E. E. Wassenaar C. Vanbavel E. van Baal M. C. (2001). Impact of current cryopreservation procedures on mechanical and functional properties of human aortic homografts. Transpl. Int. 14, 248–255. 10.1007/s001470100309 11512058

[B88] Lejay A. Delay C. Girsowicz E. Chenesseau B. Bonnin E. Ghariani M. Z. (2017). Cryopreserved cadaveric arterial allograft for arterial reconstruction in patients with prosthetic infection. Eur. J. Vasc. Endovasc. Surg. 54 (5), 636–644. 10.1016/j.ejvs.2017.07.016 28890027

[B89] Lesèche G. Penna C. Bouttier S. Joubert S. Andréassian B. (1997). Femorodistal bypass using cryopreserved venous allografts for limb salvage. Ann. Vasc. Surg. 11, 230–236. 10.1007/s100169900039 9140596

[B90] Li H. (2022). Study on the ice cellar ruins in early ancient China. Athena Trans. Soc. Sci. Hum. 2, 81–85. 10.55060/s.atssh.221230.011

[B91] Love R. (2009). Chillin’ at the symposium with plato: refrigeration in the ancient world. ASHRAE Trans. 115, 106–110.

[B92] Lovelock J. E. Bishop M. W. H. (1959). Prevention of freezing damage to living cells by dimethyl sulphoxide. Nature 183, 1394–1395. 10.1038/1831394a0 13657132

[B93] Luyet B. J. Gehenio P. M. (1940). Life and death at low temperatures. Normandy (MO): Biodynamica.

[B94] Macaulay T. B. (1882). Critical, historical, and miscellaneous essays and poems, vol. 2. Boston (MA): Estes & Lauriat, 211.

[B95] Mallis P. Katsimpoulas M. Kostakis A. Dipresa D. Korossis S. Papapanagiotou A. (2020). Vitrified human umbilical arteries as potential grafts for vascular tissue engineering. Tissue Eng. Regen. Med. 17, 285–299. 10.1007/s13770-020-00243-x 32170557 PMC7260347

[B96] Mantegazza P. (1866). Sullo sperma umano. Rendiconti R. Ist. Lomb. Sci. Lett. Cl. Sci. Mat. Nat. 3, 183–196.

[B97] Manuchehrabadi N. Gao Z. Zhang J. Ring H. L. Shao Q. Liu F. (2017). Improved tissue cryopreservation using inductive heating of magnetic nanoparticles. Sci. Transl. Med. 9 (379), eaah4586. 10.1126/scitranslmed.aah4586 28251904 PMC5470364

[B98] Marangoni A. G. Cecchini L. P. (1951). Homotransplantation of arterial segments preserved by the freeze-drying method. Ann. Surg. 134, 977–983. 10.1097/00000658-195112000-00006 14895124 PMC1802843

[B99] Mazur P. (1984). Freezing of living cells: mechanisms and implications. Am. J. Physiol. Cell Physiol. 247, C125–C142. 10.1152/ajpcell.1984.247.3.C125 6383068

[B100] Mazur P. Leibo S. P. Chu E. H. Y. (1972). A two-factor hypothesis of freezing injury. Exp. Cell Res. 71, 345–355. 10.1016/0014-4827(72)90303-5 5045639

[B101] Medynsky A. O. Holdsworth D. W. Sherebrin M. H. Rankin R. N. Roach M. R. (1998). The effect of storage time and repeated measurements on the elastic properties of isolated porcine aortas using high resolution X-ray CT. Can. J. Physiol. Pharmacol. 76, 451–456. 10.1139/cjpp-76-4-451 9795755

[B102] Mercier Dupaty C. M. J.-B. (1788). Travels through Italy in a series of letters. London: G.G.J. & J. Robinson, 100–103.

[B103] Merivaara A. Zini J. Koivunotko E. Valkonen S. Korhonen O. Fernandes F. M. (2021). Preservation of biomaterials and cells by freeze-drying: change of paradigm. J. Control Rel 336, 480–498. 10.1016/j.jconrel.2021.06.042 34214597

[B104] Merrill R. H. McLeod C. G. Jarstfer B. S. (1979). The use of lyophilized vein grafts in vascular access for chronic hemodialysis. Artif. Organs 3, 245–248. 10.1111/j.1525-1594.1979.tb01057.x 533414

[B105] Mestres C. A. Quintana E. Kopjar T. Ambrosioni J. Almela M. Fuster D. (2019). Twenty-year experience with cryopreserved arterial allografts for vascular infections. Eur. J. Cardiothorac. Surg. 55 (2), 358–365. 10.1093/ejcts/ezy263 30084901

[B106] Molnar A. Lakat T. Hosszu A. Szebeni B. Balogh A. Orfi L. (2012). Lyophilization and homogenization of biological samples improves reproducibility and reduces standard deviation in molecular biology techniques. Amino Acids 53, 917–928. 10.1007/s00726-021-02994-w 34002278 PMC8128086

[B107] Müller-Schweinitzer E. Grapow M. Konerding M. A. Zerkowski H.-R. (2005). Freezing without surrounding cryomedium preserves the endothelium and its function in human internal mammary arteries. Cryobiology 51, 54–65. 10.1016/j.cryobiol.2005.04.005 15936748

[B108] Müller-Schweinitzer E. (1992). Cryopreserved human tissue in pharmacological research. Pharmacol. Res. 25, 103–111. 10.1016/1043-6618(92)91379-u 1635889

[B109] Müller-Schweinitzer E. (2009). Cryopreservation of vascular tissue. Organogenesis 5, 97–104. 10.4161/org.5.3.9495 20046671 PMC2781088

[B110] Müller-Schweinitzer E. Tapparelli C. Victorzon M. (1986). Functional studies on human veins after storage at –190°C. Br. J. Pharmacol. 88, 685–687. 10.1111/j.1476-5381.1986.tb10251.x 2874858 PMC1916970

[B111] Müller-Schweinitzer E. (1994). “Vascular tissue preservation techniques,” in The human brain circulation. Editors Bevan R. D. Bevan J. A. (Totowa (NJ): Humana Press Inc.), 319–331.

[B112] Müller-Schweinitzer E. Mihatcsh M. J. Schilling M. Haefeli W. E. (1997). Functional recovery of human mesenteric and coronary arteries after cryopreservation at –196°C in a serum-free medium. J. Vasc. Surg. 25, 743–750. 10.1016/s0741-5214(97)70304-5 9129633

[B113] Müller-Schweinitzer E. Stulz P. Striffeler H. Haefeli W. E. (1998). Functional activity and transmembrane signaling mechanisms after cryopreservation of human internal mammary arteries. J. Vasc. Surg. 27, 528–537. 10.1016/s0741-5214(98)70328-3 9546240

[B114] Müller-Schweinitzer E. Brett W. Zerkowski H.-R. Haefeli W. E. (2000). The mechanism of cryoinjury: *in vitro* studies on human internal mammary arteries. Br. J. Pharmacol. 130, 636–640. 10.1038/sj.bjp.0703326 10821792 PMC1572092

[B115] Müller-Schweinitzer E. Striffeler H. Grussenmeyer T. Reineke D. C. Glusa E. Grapow M. T. R. (2007). Impact of freezing/thawing procedures on the post-thaw viability of cryopreserved human saphenous vein conduits. Cryobiology 54, 99–105. 10.1016/j.cryobiol.2006.11.006 17239362

[B116] Murray K. A. Gibson M. I. (2022). Chemical approaches to cryopreservation. Nat. Rev. Chem. 6, 579–593. 10.1038/s41570-022-00407-4 35875681 PMC9294745

[B117] Muss T. Lamsehchi N. Guo Y. Zhang Y. Loftin A. Bodine A. (2025). Extended subzero preservation of vascularized composite allografts using a novel bio-inspired cryoprotectant: evaluating functional recovery through an orthotopic forelimb transplantation model. AJT 25 (8), S564. 10.1016/j.ajt.2025.07.1316

[B118] Năstase G. Botea F. Beşchea G.-A. Câmpean Ș.-I. Barcu A. Neacşu I. (2023). Isochoric supercooling organ preservation system. Bioengineering 10 (8), 934. 10.3390/bioengineering10080934 37627819 PMC10451689

[B119] Needham T. A. (1743). A letter from Mr. Turbevil Needham, to the president: concerning certain chalky tubulous concretions, called malm: with some microscopical observations on the farina of the red lily, and of worms discovered in smutty corn. Philos. Trans. 42, 634–641.

[B120] Ochsner J. L. Lawson J. D. Eskind S. J. Mills N. L. DeCamp P. T. (1984). Homologous veins as an arterial substitute: long-term results. J. Vasc. Surg. 1, 306–313. 10.1067/mva.1984.avs0010306 6481879

[B121] Ozgur O. S. Namsrai B.-E. Pruett T. L. Bischof J. C. Toner M. Finger E. B. (2023). Current practice and novel approaches in organ preservation. Front. Transpl. 2, 1156845. 10.3389/frtra.2023.1156845 38993842 PMC11235303

[B122] O’Leary S. A. Doyle B. J. McGloughlin T. M. (2014). The impact of long term freezing on the mechanical properties of porcine aortic tissue. J. Mech. Behav. Biomed. Mater 37, 165–173. 10.1016/j.jmbbm.2014.04.015 24922621

[B123] Pascual G. Garcia-Honduvilla N. Rodriguez M. Turégano F. Bujan J. Bellón J. M. (2001). Effect of the thawing process on cryopreserved arteries. Ann. Vasc. Surg. 15, 619–627. 10.1007/s100160010130 11769142

[B124] Pasquinelli G. Foroni L. Buzzi M. Tazzari P. L. Vaselli C. Mirelli M. (2006). Smooth muscle cell injury after cryopreservation of human thoracic aortas. Cryobiology 52, 309–316. 10.1016/j.cryobiol.2005.12.004 16458877

[B125] Pegg D. E. Wusteman M. C. Boylan S. (1997). Fractures in cryopreserved elastic arteries. Cryobiology 34, 183–192. 10.1006/cryo.1996.1997 9130389

[B126] Pegg D. E. (2015). “Principles of cryopreservation,” in Cryopreservation and freeze-drying protocols. (New York: Springer Science Business Media), 3–19.

[B127] Pierce E. C. Gross R. E. Bill A. H. Merrill K. (1949). Tissue-culture evaluation of the viability of blood vessels stored by refrigeration. Ann. Surg. 129, 333–348. 10.1097/00000658-194903000-00006 PMC1514023 17859314

[B128] Polge C. Smith A. U. Parkes A. S. (1949). Revival of spermatozoa after vitrification and dehydration at low temperatures. Nature 164, 666. 10.1038/164666a0 18143360

[B129] Power H. (1664). Experimental philosophy in three books: containing new experiments microscopical, mercurial, magnetical. London: T. Roycroft, 35.

[B130] Pukacki F. Jankowski T. Gabriel M. Oszkinis G. Krasinski Z. Zapalski S. (2000). The mechanical properties of fresh and cryopreserved arterial homografts. Eur. J. Vasc. Endovasc. Surg. 20, 21–24. 10.1053/ejvs.2000.1120 10906292

[B131] Rob C. G. (1954). The preservation of arterial grafts by freeze-drying. Proc. Roy. Soc. Med. 47, 368–370. 10.1177/003591575404700516 13167056 PMC1918858

[B132] Rall W. F. Fahy G. M. (1985). Ice-free cryopreservation of mouse embryos at –196°C by vitrification. Nature 313, 573–575. 10.1038/313573a0 3969158

[B133] Reeves T. R. Cezeaux J. L. Sackman J. E. Cassada D. C. Freeman M. B. Stevens S. L. (1997). Mechanical characteristics of lyophilized human saphenous vein valves. J. Vasc. Surg. 26, 823–828. 10.1016/s0741-5214(97)70096-x 9372821

[B134] Ren Z.-Y. Wu Q. Pan B. Liu J.-Z. He Q. Lang R. (2023a). Effects of different preservation schemes on isolated rat artery. J. Cell Mol. Med. 27, 2362–2371. 10.1111/jcmm.17822 37357501 PMC10424285

[B135] Ren Z.-Y. Pan B. Wang F.-F. Lyu S.-C. He Q. (2023b). Effects of different preservation methods of human iliac veins. Cell Tissue Bank. 24, 571–582. 10.1007/s10561-022-10055-z 36441442

[B136] Rosset E. Friggi A. Novakovitch G. Rolland P.-H. Rieu R. Pellisier J.-F. (1996). Effects of cryopreservation on the viscoelastic properties of human arteries. Ann. Vasc. Surg. 10, 262–272. 10.1007/BF02001892 8792995

[B137] Rubinsk B. Perez P. A. Carlson M. E. (2005). The thermodynamic principles of isochoric cryopreservation. Cryobiology 50, 121–138. 10.1016/j.cryobiol.2004.12.002 15843002

[B138] Sandler I. (1973). The re-examination of Spallazani’s interpretation of the role of spermatic animalcules in fertilization. J. Hist. Biol. 6, 193–223. 10.1007/BF00127608 11609721

[B139] Santana D. B. Armentano R. L. Zocalo Y. Pérez Cámpos H. Cabrera Fischer E. I. Graf S. (2007). Functional properties of fresh and cryopreserved carotid and femoral arteries, and of venous and synthetic grafts: comparison with arteries from normotensive and hypertensive patients. Cell Tissue Bank. 8, 43–57. 10.1007/s10561-006-9000-5 16826454

[B140] Sasson J. M. (1984). Thoughts of Zimri-Lim. Bib Arch. 47, 110–120. 10.2307/3209891

[B141] Schulte-Wrede S. Staudacher M. Weissenhofer W. Wetzstein R. (1975). Lyophilized veins studied by scanning electron microscopy. Eur. Surg. Res. 7, 120–128. 10.1159/000127798 1140209

[B142] Seghers M. J. Longacre J. J. (1964). Paul Bert and his animal grafts. Plast. Reconstr. Surg. 33, 178–186. 10.1097/00006534-196402000-00009 14120252

[B143] Sellers M. T. Haustein S. V. McGuire B. M. Jones C. Bynon J. S. Diethelm A. G. (2002). Use of preserved vascular homografts in liver transplantation: hepatic artery aneurysms and other complications. Am. J. Transpl. 2, 471–475. 10.1034/j.1600-6143.2002.20513.x 12123215

[B144] Smardencas A. Birchall I. (2011). Morphological changes in the ovine carotid artery wall induced by cold storage. Cell Transpl. 20, 1603–1620. 10.3727/096368910X564517 21396174

[B145] Smith A. U. (1958). The resistance of animals to cooling and freezing. Biol. Rev. 33, 197–253. 10.1111/j.1469-185x.1958.tb01446.x

[B146] Song Y. C. Hunt C. J. Pegg D. E. (1994). Cryopreservation of the common carotid artery of the rabbit. Cryobiology 31, 317–329. 10.1006/cryo.1994.1038 7523024

[B147] Song Y. C. Pegg D. E. Hunt C. J. (1995). Cryopreservation of the common carotid artery of the rabbit: optimization of dimethyl sulfoxide concentration and cooling rate. Cryobiology 32, 405–421. 10.1006/cryo.1995.1040 7587281

[B148] Song Y. C. Khirabadi B. S. Lightfoot F. Brockbank K. G. M. Taylor M. J. (2000a). Vitreous cryopreservation maintains the function of vascular grafts. Nat. Biotechnol. 18, 296–299. 10.1038/73737 10700144

[B149] Song Y. C. Hagen P.-O. Lightfoot F. Taylor M. J. Smith A. C. Brockbank K. G. M. (2000b). *In vivo* evaluation of the effects of a new ice-free cryopreservation process on autologous vascular grafts. J. Invest. Surg. 13, 279–288. 10.1080/08941930050206300 11071564

[B150] Soo E. Marsh C. Steiner R. Stocks L. McKay D. B. (2020). Optimizing organs for transplantation; advancements in perfusion and preservation method. Transpl. Rev. 34, 100514. 10.1016/j.trre.2019.100514 31645271 PMC6905200

[B151] Spallanzani L. (1776). Opuscoli di Fisica Animale, e Vegetabile. Volume Secondo. Opuscolo II: osservazioni, e Sperienze Intorno ai Vermicelli Spermatici dell’ Uomo, e degli Animali, Vol. 2. Modena: Societa Tipografica.

[B152] Stemper B. D. Yoganandan N. Stineman M. R. Gennarelli T. M. Baisden J. L. Pintar F. A. (2007). Mechanics of fresh, refrigerated, and frozen arterial tissue. J. Surg. Res. 139, 236–242. 10.1016/j.jss.2006.09.001 17303171

[B153] Sztein J. M. Takeo T. Nakagata N. (2018). History of cryobiology, with special emphasis in evolution of mouse sperm cryopreservation. Cryobiology 82, 57–63. 10.1016/j.cryobiol.2018.04.008 29660317

[B154] Tabiei A. Cifuentes S. Colglazier J. J. Shuja F. Kalra M. Mendes B. C. (2024). Cryopreserved arterial allografts vs autologous vein for arterial reconstruction in infected fields. J. Vasc. Surg. 79 (4), 941–947. 10.1016/j.jvs.2023.12.011 38101708

[B155] Taylor M. J. Weegman B. P. Baicu S. C. Giwa S. E. (2019). New approaches to cryopreservation of cells, tissues, and organs. Transfus. Med. Hemother 46, 197–215. 10.1159/000499453 31244588 PMC6558330

[B156] Thakrar R. R. Patel V. P. Hamilton G. Fuller B. J. Seifalian A. M. (2006). Vitreous cryopreservation maintains the viscoelastic property of human vascular grafts. FASEB J. 20, 874–881. 10.1096/fj.05-5304com 16675845

[B157] Thatte H. S. Khuri S. F. (2001). The coronary artery bypass conduit: I. Intraoperative endothelial injury and its implication on graft patency. Ann. Thorac. Surg. 72, S2245–S2252. 10.1016/s0003-4975(01)03272-6 11789848

[B158] Timaran C. H. Stevens S. L. Freeman M. B. Goldman M. H. (2002). Infrainguinal bypass grafting using lyophilized saphenous vein allografts for limb salvage. Cardiovasc Surg. 10, 315–319. 10.1016/s0967-2109(02)00025-x 12359400

[B159] Toner M. Cravalho E. G. Karel M. (1990). Thermodynamics and kinetics of intracellular ice formation during freezing of biological cells. J. Appl. Phys. 67, 1582–1593. 10.1063/1.345670

[B160] Toto F. Torre T. Turchetto L. Lo Cicero V. Soncin S. Klersy C. (2022). Efficacy of intraoperative vein graft storage solutions in preserving endothelial integrity during coronary artery bypass surgery. J. Clin. Med. 11, 1093. 10.3390/jcm11041093 35207364 PMC8877698

[B161] Touma J. Cochennec F. Parisot J. Fialaire Legendre A. Becquemin J.-P. Desgranges P. (2014). *In situ* reconstruction in native and prosthetic aortic infections using cryopreserved arterial allografts. Eur. J. Vasc. Endovasc. Surg. 48, 292–299. 10.1016/j.ejvs.2014.04.023 24923233

[B162] Trump B. F. Young D. E. Arnold E. A. Stowell R. E. (1965). Effects of freezing and thawing on the structure, chemical constitution, and function of cytoplasmic structures. Fed. Proc. 24, S144–S168. 14314561

[B163] Tsakok M. Montgomery-Taylor S. Tsakok T. (2012). Storage of saphenous vein grafts prior to coronary artery bypass grafting: is autologous whole blood more effective than saline in preserving graft function? Interact. CardioVasc Thorac. Surg. 15, 720–725. 10.1093/icvts/ivs275 22753436 PMC3445367

[B164] Valente S. Alviano F. Ciavarella C. Buzzi M. Ricci F. Tazzari P. L. (2014). Human cadaver multipotent stromal/stem cells isolated from arteries stored in liquid nitrogen for 5 years. Stem Cell Res. Ther. 5, 8. 10.1186/scrt397 24429026 PMC4055119

[B165] Van Leewenhoeck A. (1678). Observations, communicated to the publisher by Mr. van Leewenhoeck, in a Dutch letter of the 9th of October 1676. Here English’d: concerning little animals by him observed in rain-well-sea- and snow water; as also in water wherein pepper had lain infused. Philos. Trans. 12, 821–831. 10.1098/rstl.1677.0003

[B166] Venkatasubramanian R. T. Grassl E. D. Barocas V. H. Lafontaine D. Bischof J. C. (2006). Effects of freezing and cryopreservation on the mechanical properties of arteries. Ann. Biomed. Eng. 34, 823–832. 10.1007/s10439-005-9044-x 16619131

[B167] Watson P. F. (1979). The preservation of semen in mammals. Oxf. Rev. Reprod. Biol. 1, 283–350.

[B168] Weber T. R. Dent T. L. Lindenauer S. M. Allen E. Weatherbee L. Spencer H. H. (1975a). Viable vein graft preservation. J. Surg. Res. 18, 247–255. 10.1016/0022-4804(75)90148-1 166254

[B169] Weber T. R. Lindenauer S. M. Dent T. L. Allen E. Salles C. A. Weatherbee L. (1975b). Long-term patency of vein grafts preserved in liquid nitrogen in dimethyl sulfoxide. Ann. Surg. 184, 709–712. 10.1097/00000658-197612000-00008 999347 PMC1345412

[B170] Weiss S. Bachofen B. Widmer M. K. Makaloski V. Schmidli J. Wyss T. R. (2021). Long-term results of cryopreserved allografts in aortoiliac graft infections. J. Vasc. Surg. 74 (1), 268–275. 10.1016/j.jvs.2020.12.070 33348005

[B171] Wille T. de Groot H. Rauen U. (2008). Improvement of the cold storage of blood vessels with a vascular preservation solution. Study in porcine aortic segments. J. Vasc. Surg. 47, 422–431. 10.1016/j.jvs.2007.09.048 18164170

[B172] Winkler B. Reineke D. Heinisch P. P. Schönhoff F. Huber C. Kadner A. (2016). Graft preservation solutions in cardiovascular surgery. Interact. CardioVasc Thorac. Surg. 23, 300–309. 10.1093/icvts/ivw056 27068248

[B173] Wood T. H. (1956). “Lethal effects of high and low temperatures on unicellular organisms,” in Advances in biological and medical physics, vol. 4. Editors Lawrence J. H. Tobias C. A. (New York: Academic Press), 119–166. 10.1016/b978-1-4832-3110-5.50008-x 13354510

[B174] Woodward L. C. Antoniades C. Taggart D. P. (2016). Intraoperative vein graft preservation: what is the solution? Ann. Thorac. Surg. 102, 1736–1746. 10.1016/j.athoracsur.2016.05.097 27624295

[B175] Wright J. C. (2001). Cryptobiosis 300 years on from van Leuwenhoek: what have we learned about tardigrades? Zool. Anz. 240, 563–582. 10.1078/0044-5231-00068

[B176] Zatschler B. Dieterich P. Müller B. Kasper M. Rauen U. Deussen A. (2009). Improved vessel preservation after 4 days of cold storage: experimental study in rat arteries. J. Vasc. Surg. 50, 397–406. 10.1016/j.jvs.2009.04.064 19631875

[B177] Zhang A. Cheng S. Gao D. Xu L. X. (2005). Thermal stress study of two different artery cryopreservation methods. CryoLetters 26, 113–120. 15897963

